# Molecular Characterization and Clinicopathological Findings of *Mycoplasma pogonae* Infection in Captive Central Bearded Dragons (*Pogona vitticeps*)

**DOI:** 10.3390/ani16010048

**Published:** 2025-12-24

**Authors:** Tithipong Plangsangmas, Alexandra Burne, Eliana De Luca, Emi Sasaki, Jose Cesar Menk Pinto Lima, Kelsey Konopka, Mary B. Brown, Javier G. Nevarez

**Affiliations:** 1 Department of Veterinary Clinical Sciences, Louisiana State University School of Veterinary Medicine, Baton Rouge, LA 70803, USA; tplangsangmas@lsu.edu; 2 Department of Science, Technology, and Innovation, Faculty of Science, Chulabhorn Royal Academy, Lak Si, Bangkok 10210, Thailand; 3 Department of Infectious Diseases and Immunology, University of Florida College of Veterinary Medicine, Gainesville, FL 32608, USA; abg88@missouri.edu (A.B.); kelsey.konopka@ufl.edu (K.K.); mbbrown@ufl.edu (M.B.B.); 4 Louisiana Animal Diagnostic Disease Laboratory, Department of Pathobiological Sciences, Louisiana State University School of Veterinary Medicine, Baton Rouge, LA 70803, USA; edeluca@lsu.edu (E.D.L.); emi@lsu.edu (E.S.); cmenk2@lsu.edu (J.C.M.P.L.)

**Keywords:** *Bearded dragon*, *Mycoplasma pogonae*, *Mycoplasmosis*, *Pogona vitticeps*, polymerase chain reaction, sequence analysis, *Squamata*

## Abstract

From August to November 2023, a disease outbreak occurred in a research colony of 33 mixed-sex, five-month-old central bearded dragons obtained from a private breeder in the USA. Clinical presentation consisted primarily of dehydration, lethargy, inappetence, weight loss, neck extension, sporadic open mouth breathing indicating dyspnea, and sudden death. A total of six deaths occurred during this period. Pathological investigation followed by molecular diagnostics and whole-genome sequencing revealed *Mycoplasma pogonae* as the cause of disease. Pneumonia (5/6) was diagnosed from necropsy. Types of pneumonia included histiocytic (1/5), combined histiocytic and lymphocytic (2/5), histiocytic and granulocytic (1/5), and heterophilic pneumonia (1/5). Our results confirmed that *M. pogonae* infection is associated with sudden mortality in a colony of captive central bearded dragons in the USA and that *M. pogonae* has a potential pathogenic role as a primary infectious agent.

## Simple Summary

Between August and November 2023, a disease outbreak affected a research colony of 33 juvenile central bearded dragons in the USA. The dragons exhibited clinical signs including dehydration, lethargy, weight loss, difficulty breathing (dyspnea), and sudden death, resulting in six deaths. A full investigation, using molecular diagnostics and whole-genome sequencing, identified *Mycoplasma pogonae* as the cause. Pathological findings confirmed pneumonia in all affected animals, with various inflammatory cell types observed (histiocytic, lymphocytic, granulocytic, and heterophilic). This study suggests that *M. pogonae* has the ability to cause severe illness and mortality in captive bearded dragons.

## 1. Introduction

Central or inland bearded dragons (*Pogona vitticeps*) are considered the most popular pet reptile and have been bred in captivity with great success [1]. Nevertheless, optimal husbandry conditions are not always achieved for reptiles in captivity. Inappropriate diet, temperature, humidity, and hygiene can compromise health and immunity, allowing pathogens to cause clinical disease [2]. Bacterial infections, in particular, tend to be secondary to improper husbandry or as coinfections with other diseases such as viral infections [3].

*Mycoplasma* spp. are common etiologic agents of upper respiratory tract disease (URTD) in reptiles and have been reported as either primary [4,5,6,7,8,9] or secondary pathogens with coinfections [10,11]. Recently, the taxonomy of the genus *Mycoplasma* (*M.*) has been revised [12,13,14]; however, in this article, we will refer to the traditional names of genera and species as supported by more recent comprehensive genomic analyses [15]. Clinical signs of mycoplasmosis in reptiles include dyspnea, anorexia, serous-to-mucopurulent nasal and ocular discharge, edematous and erythematous conjunctiva, periocular and palpebral edema, tracheitis, pneumonia, polyserositis, and polyarthritis [16,17]. Subclinical infection may also occur, leading to asymptomatic carriers.

*Mycoplasma pogonae* was identified by 16S rRNA gene PCR and proposed as a provisional novel species in a single bearded dragon co-infected with helodermatid adenovirus 2 [18]. Another report of mycoplasma infection in bearded dragons from Hungary described clinicopathological characteristics, but the infection was never confirmed as *M. pogonae* [19]. Here, we describe the clinical and pathological findings in a disease outbreak in central bearded dragons naturally infected with *M. pogonae.* We also provide foundational support based on whole-genome sequencing data of *M. pogonae* L2313072 as the type strain of the new species. To date, there are no additional reports of *M. pogonae* in the literature, and this is the first report of direct axenic clinical isolation of *M. pogonae* from bearded dragons.

The objectives of this study were (1) to report an outbreak of *M. pogonae* infection in a research colony of central bearded dragons, (2) to describe the pathological changes related to *M. pogonae* infection in central bearded dragons, and (3) to confirm *M. pogonae* infection via molecular characterization.

## 2. Materials and Methods

### 2.1. Overview and Animals

**Figure 1:**
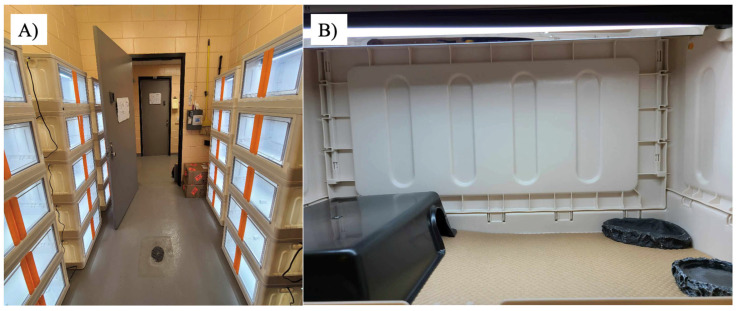
Bearded dragon housing conditions. (**A**) Room with container stacks and (**B**) inside of each container, which consists of a UVB light on the top, a hide box, food tray, and water tray.

Gross and histopathological data from six captive central bearded dragons that died during August–November 2023 (five to seven months old) were obtained from the Louisiana Animal Disease Diagnostic Laboratory (LADDL) at the Louisiana State University School of Veterinary Medicine, USA. All animals were part of a research colony of 33, five-month-old, mixed sex, central bearded dragons obtained from a private breeder in the USA. Animals were kept individually in 66 × 38 × 37 cm plastic enclosures (Figure 1). Their diet consisted solely of Repashy Beardie Buffet^®^ (Repashy Ventures, Inc., Oceanside, CA, USA) given daily ad libitum. Clean water offered in a shallow bowl was changed daily. A single fluorescent UVB bulb (Arcadia Pro T5 6%, Arcadia Reptile, Mepal, Cambridgeshire, UK) was present inside each cage, mounted on an aluminum reflector hood, 30 cm from the bottom of the cage with no material present between the bulb and the animals. The UV index at the bottom of the cage, directly under the bulb, was 5.3 with this setup. The UVB photoperiod was 8 h per day (7:00 a.m. to 3:00 p.m.). A single plastic hideout (33 × 23 × 8 cm) allowed the animal to transition between dark and light conditions voluntarily. The daytime temperature gradient ranged between 85 and 93 degrees Fahrenheit (29.4–33.9 degrees Celsius) through the control of room temperature without the use of an additional heat source, such as a basking bulb. The nighttime temperature gradient ranged between 78 and 85 degrees Fahrenheit (25.56–29.4 degrees Celsius). Humidity ranged between 30 and 50%.

### 2.2. Pathological Investigation

Deceased animals consisted of three males and three females. A complete necropsy was performed on each animal as per the standard protocol of LADDL. Sections of all major organs were fixed in 10% neutral-buffered formalin, routinely processed into paraffin blocks, sectioned at a 4 µm thickness, and stained with hematoxylin and eosin. The heads were decalcified in 15% formic acid, and multiple serial transverse sections were obtained and similarly processed.

### 2.3. Mycoplasma Culture

Fresh lung or tracheal tissues and swabs were collected at necropsy from 5 of the 6 animals. Swabs were placed in universal viral transport medium without antibiotics. Tissues and swabs were frozen at −80 °C and shipped to the Mycoplasma Research Diagnostic Laboratory, Department of Infectious Diseases and Immunology, University of Florida College of Veterinary Medicine, USA. From case 1, no tissue from necropsy was available for culture, but formalin-fixed paraffin-embedded (FFPE) scrolls were positive for *Mycoplasma* spp. by direct PCR. For frozen tissue collected at necropsy, tissues were minced in 500 µL of SP4 broth medium containing 0.5% glucose and 0.21% arginine (SP4 G+A). Ten-fold serial dilutions were made in SP4+GA broth, 20 µL of each dilution was plated onto SP4 G+A agar, and plates were incubated at 30 °C and 5% CO_2_ for 5 days. Lung swabs (*n* = 2) were placed in SP4 G+A broth, serially diluted, and plated as described for tissue samples. A direct PCR of tissues obtained at necropsy before October (*n* = 4) were positive for *Mycoplasma* spp. Because of the success in direct culture, no PCR from tissues was performed on animals necropsied after September (*n* = 2). Any remaining minced tissue was collected in a sterile 1 mL tube and frozen at −80 °C. Broth dilutions were incubated at 30 °C for 5 days. All positive broth cultures (*n* = 5) were confirmed as *M. pogonae* by PCR targeting the 16S rRNA and *uvrA* genes. Agar plates were incubated similarly and checked for the development of colonies with characteristic ‘fried-egg’ morphology. Positive cultures were collected in 1 mL aliquots and stored at −80 °C.

### 2.4. Molecular Characterization

#### 2.4.1. DNA Extraction and Conventional PCR Detection of *Mycoplasma* spp.

**Figure 2:**
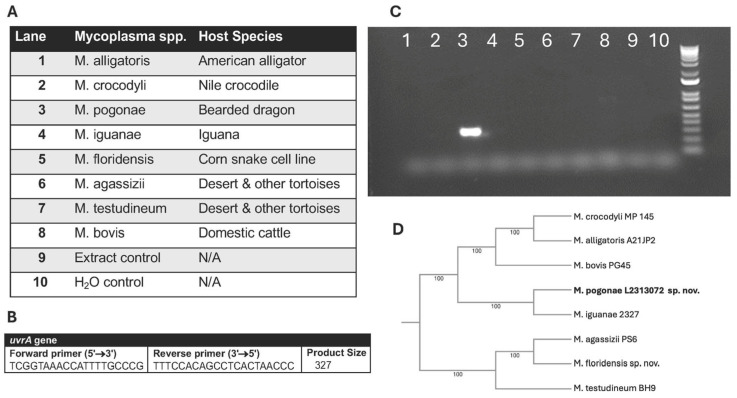
Confirmation of *M. pogonae* by PCR using species-specific primers for the *uvrA* gene. (**A**) Lane location for *Mycoplasma* spp. and reptilian host. (**B**) *uvrA* primers. (**C**) Gel of PCR results showing the detection of *M. pogonae* (lane 3) and absence of a reaction with other *Mycoplasma* spp. from reptilian hosts. (**D**) 16S Phylogeny of *Mycoplasma* spp. from reptilian hosts.

DNA was extracted from 200 μL of broth cultures using a commercially available DNA extraction kit (PureLink™ Genomic DNA Mini Kit, Invitrogen, Waltham, MA, USA) following the manufacturer’s instructions. PCR amplification was performed using established 16S rRNA primers and cycle conditions for the detection of *Mycoplasma* spp. [20]. Purified amplicons were submitted to Lone Star Labs (College Station, TX, USA) for Sanger sequencing, followed by BLASTN (version 2.17.0) analysis against NCBI databases. Additionally, all positive samples were confirmed as *M. pogonae* by PCR targeting the *uvrA* gene using primers that did not cross-react with other known mycoplasmas from reptilian hosts (Figure 2).

#### 2.4.2. Whole-Genome Sequencing

*M. pogonae* strain L2313072 isolated from the lung was chosen for whole-genome sequencing (WGS). For genomic DNA extraction, 50 mL of a mid-log phase culture was spun at >12,000× *g* for 60 min. Pellets were washed in 1× phosphate-buffered saline (PBS) three times. After resuspension in 2 mL PBS, samples were aliquoted into sterile 1.5 mL tubes and an appropriate volume of TELT buffer (50 mM Tris pH 8.0, 62.5 mM EDTA pH 8.0, 2.4 M LiCl, 4% Triton X-100) was added until pellets were lysed via gentle rocking. An equal volume of phenol–chloroform isoamyl was added, and the sample was spun at >12,000× *g* for 10 min. The aqueous phase was removed, and an equal volume of ice-cold isopropanol was added to precipitate total nucleic acid. The precipitate was pelleted as before and washed in 70% ethanol. Tubes were air dried for 3 min, and total nucleic acid was resuspended in nuclease-free water. All reagents and kits were obtained from Thermo Fisher Scientific, Waltham, MA, USA. The DNA was quantified via a Qubit^TM^ Broad Range Assay and submitted to SeqCenter (Pittsburgh, PA, USA) for Illumina Nova Seq 6000 sequencing. Quality control and adapter trimming were performed with bcl2fastq (https://support.illumina.com/sequencing/sequencing_software/bcl2fastq-conversion-software.html accessed on 12 September 2025). Short read assembly was performed with Unicycler (v0.5.1) [21]. Assembly statistics were recorded with QUAST (v5.3.0) [22] and annotation was performed with Prokka (v1.15.6) [23]. Comparison of the *M. pogonae* genome sequence (NCBI BioProject ID: PRJNA1333947) with known Mollicutes species for 16S rRNA and proteomic phylogeny as well as average nucleotide identity (ANI) was performed using the Type (Strain) Genome Server [24,25,26]. Genomes were compared for ANI using OrthoANI [26,27]. Websites for the programs used in these analyses can be found at https://www.ezbiocloud.net/tools/orthoaniu (accessed on 12 September 2025) and https://tygs.dsmz.de (accessed on 12 September 2025).

## 3. Results

### 3.1. Clinicopathological Findings

Detailed information of all the bearded dragon deaths during August–November 2023, including signalments, culture isolation and PCR results of *M. pogonae*, and clinicopathological findings, is summarized in Table 1.

Early clinical signs included lethargy, inappetence, weight loss, dehydration, and blepharospasm. Animals were treated with lactate ringer’s solution of 30 mL/kg/day subcutaneously. Only one animal presented signs for more than 24 h; the remaining five died within 24 h after initial clinical signs.

**Figure 3:**
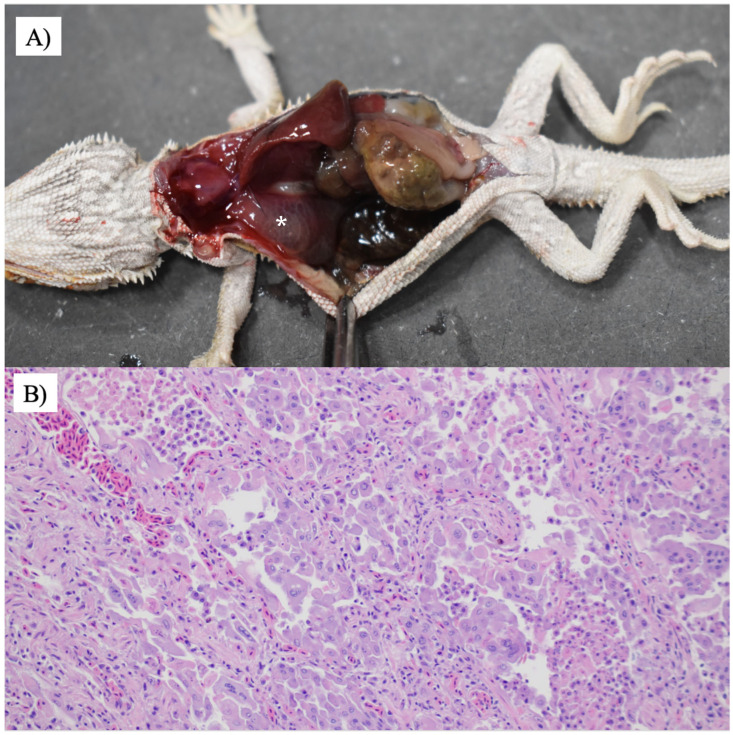
Mycoplasmosis in a central bearded dragon. (**A**) The lungs are diffusely dark pink and contain large amounts of pink-tinged translucent fluid (white asterisk). (**B**) The faveoli are frequently lined by a flat-to-cuboidal epithelium. There is marked type II pneumocyte hyperplasia with significant karyomegaly and marginated chromatin. The faveolae contain a large number of free-floating foamy macrophages, heterophils, and cellular debris. Hematoxylin and eosin staining was used. 200×.

Emaciation, lethargy, and dehydration were found in all six animals, while pneumonia was diagnosed in five animals. Representative gross lesions and histological lesions of the lungs are shown in Figure 3. Types of pneumonia included histiocytic (1/5), combined histiocytic and lymphocytic (2/5), histiocytic and granulocytic (1/5), and heterophilic pneumonia (1/5). All animals with pneumonia (5/5) had *M. pogonae* found in the lung (*n* = 3) or trachea (*n* = 1; lung samples not submitted) by either culture (*n* = 4) or PCR (*n* = 1, tissues not available for culture). Signs of URTD [conjunctivitis, nasopharyngitis, rhinitis, stomatitis, pharyngitis, or dacryocystitis] were found in two animals. Intranuclear inclusion bodies were found in enterocytes, hepatocytes, and harderian gland epithelial cells in case 5 and pneumocytes and colonic epithelial cells in case 4. Parasitism was found in two animals including a large number of *Isospora* sp. and oxyurid ova seen from the colon in one animal and numerous intestinal parasites suspected to be *Balantidium* spp. in a second animal.

### 3.2. Mycoplasma Culture

**Figure 4:**
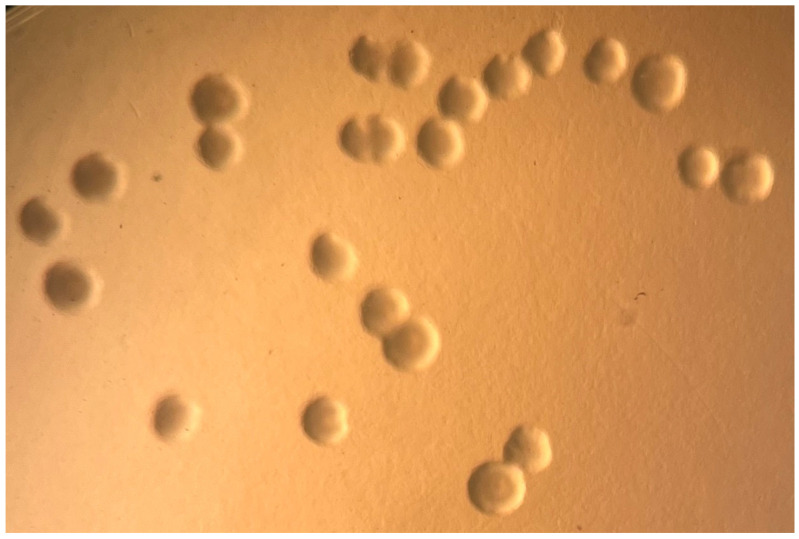
Growth of *M. pogonae* from frozen lung tissue showing classical fried egg colonial morphology on SP4+GA agar. 10×.

After 5 days of incubation at 30 °C, pinpoint colonies of approximately 0.5 mm in diameter and displaying a typical fried egg appearance on SP4 G+A agar (Figure 4) were observed on cultures from at least one organ (Table 1) for five animals. One animal had only FFPE scrolls submitted and therefore could not be cultured, but this animal was positive by direct PCR of the 16S RRNA gene. For animals with pneumonia, *M. pogonae* was isolated from the lung of all cases (2, 3, and 6) for which lung tissues were submitted. Case 4 had *M. pogonae* isolated from the trachea, but no lung sample was submitted. Only formalin-fixed tissues were available for case 1, and *M. pogonae* was detected in the lung by PCR. The animal from case 5 did not have pneumonia and was negative by culture for *M. pogonae* in lung tissue but did have *M. pogonae* isolated from a swab of the lung tissue. PCR was not performed on samples from case 5.

### 3.3. Molecular Characterization

**Figure 5:**
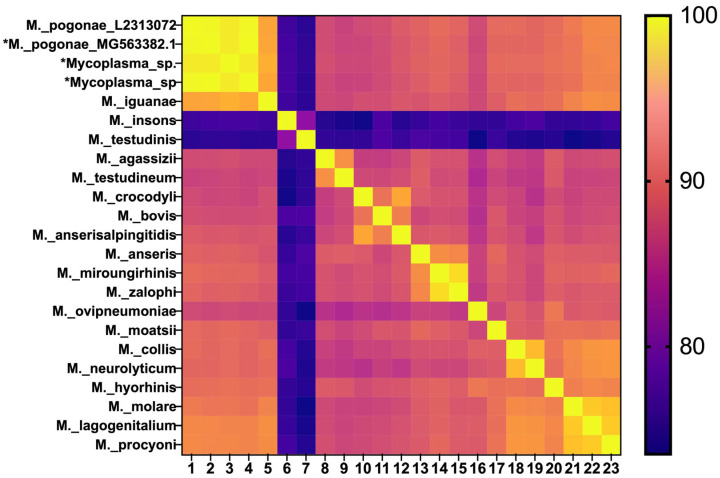
Heat map for percent identity of 16S rRNA sequences. *M. pogonae* L2313072 from this study [lane 1 and 16S sequences from the USA (lane 2) and Europe (lanes 3 and 4) are shown on the top left (asterisk)].

A single colony from each animal was expanded and subjected to molecular characterization by PCR amplification of the 16S rRNA gene using generic conserved primers complementary to terminal sequence sense strand nucleotides (nt) 11–30 (5′-AGAGTTTGATCCTGGCTCAGGA-3′) and a Mycoplasma genus-specific region anti-sense strand nt 1055 to 1031 (5′-TGCACCATCTGTCACTCTGTTAACCTC-3′), as previously described [20]. Purified amplicons were submitted to Lone Star Labs (College Station, TX, USA) for Sanger sequencing. Sequence data covered the majority of the conserved and variable regions of the gene, resulting in a 1530 bp amplicon. The 16S rRNA gene products from the *M. pogonae* isolates in this study were identical. Furthermore, *M. pogonae* L2313072 had >99% sequence identity to the 16S rRNA sequences reported for an uncultivated *Mycoplasma* sp. from the lung of a bearded dragon in the USA [18], as well as an unnamed *Mycoplasma* sp. from the nasal cavity (strain 3520, OR879222.1) and fistula (strain 1620, MT735161) of bearded dragons in Europe (Figure 5).

**Figure 6:**
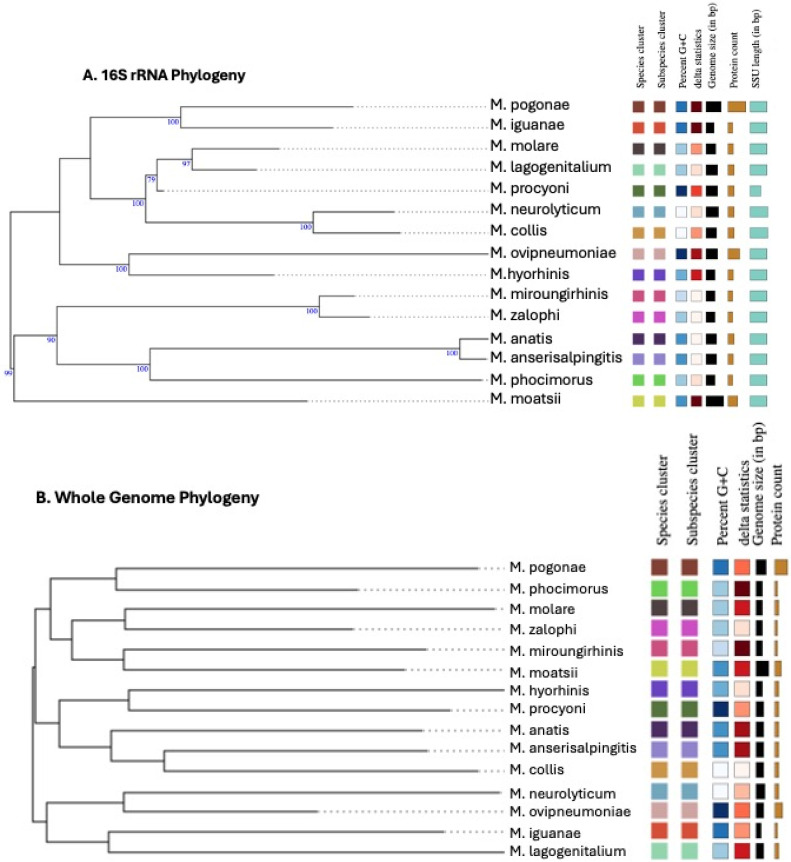
Phylogenetic trees based on 16S rRNA sequence and whole-genome comparison were constructed using the Type (Strain) Genome Server. Both trees support that *M. pogonae* is a new species. Species status is denoted by different colors. (**A**) 16S rRNA phylogenetic tree. (**B**) Whole-genome comparison phylogenetic tree. Genomes were compared for average nucleotide identity (ANI) using OrthoANI, predicted digital DNA/DNA hybridization (dDDH). See Table 2. Websites for the programs used in these analyses are https://www.ezbiocloud.net/tools/orthoaniu and https://tygs.dsmz.de.

Analysis of phylogenetic trees based on the 16S rRNA sequence (Figure 6A) and whole-genome comparison at both the ANI (Figure 6B) and digital DNA/DNA hybridization (dDDH) (Table 2) levels confirmed that *M. pogonae* met the established molecular criteria for a new species [28,29,30].

Table 2 provides the dDDH values and confidence intervals (CIs) for the whole genome sequence of *M. pogonae* compared with other *Mycoplasma* spp. For all three formulas, the differences were significantly lower than the 70% species boundary. Three different Genome Blast Distance Phylogeny (GBDP) formulas are used to calculate the genome-to-genome distance calculator (GGDC). For all formulas, high-scoring segment pairs (HSPs) are identified during alignment. The results in Table 2 reflect the results for the independent alignment of *M. pogonae* with the genome of each Type Strain. Formula d0 is the length of all HSPs divided by the total genome. Formula d4 is the sum of all identities found in HSPs divided by the overall HSP length. Formula d6 is the sum of all identities found in HSPs divided by the total genome length. Formula d4 is independent of genome length and is thus robust against the use of incomplete draft genomes. Because mycoplasma genomes in GenBank were acquired using Illumina sequencing, the genomes are generally in multiple contigs rather than a single circular chromosome, making formula d4 likely the most reliable. Importantly, for all three formulas used, the dDDH % identity ranged from 12.7 to 27.1, which is well below the accepted species boundary of 70% [24,28,30,31].

## 4. Discussion

*M. pogonae* was first reported by molecular detection using pan-mycoplasma 16S rRNA primers with sequencing and phylogenetic analysis from fresh frozen lung tissues of a single, four-year-old, male bearded dragons that succumbed to respiratory illness characterized by dyspnea, increased respiratory sounds, and mucoid oral discharge [18]. Histological features in this animal included heterophilic and lymphocytic interstitial pneumonia, with intranuclear inclusions, pneumocyte hyperplasia, and squamous metaplasia of the respiratory epithelium [18]. Ziszisz et al. [19] diagnosed mycoplasmosis in an adult central bearded dragon with signs of lethargy, inappetence, and weight loss for 3 weeks by a conventional universal mycoplasma PCR system specific for the 16S rRNA–23S rRNA intergenic region. The animal also had respiratory signs including increased clear nasal discharge and mucoid foamy discharge from the oral cavity. Necropsy revealed acute rhinitis, tracheitis with epithelial degeneration, and pneumonia accompanied by muco-serous fluid accumulation. The clinical signs of the affected animals in our study group consisted primarily of dehydration, lethargy, inappetence, and weight loss, but only one animal had respiratory signs that manifested as cervical distension and sporadic open mouth breathing, indicating dyspnea. The signs reported in our study resemble Ziszisz et al.’s [19] report; however, the respiratory signs were not as pronounced. The animals in our study were younger and smaller, and we can infer that their immunity is weaker. It is speculated that the disease presented as more acute in our study due to the animals being immunocompromised from inadequate husbandry. In our study population, pneumonia was diagnosed in all *M. pogonae*-positive animals. Two of the animals had viral inclusion bodies found histologically in enterocytes, hepatocytes, pneumocytes, harderian gland epithelial cells, and colonic epithelial cells, while the helodermatid adenovirus 2 infection from the previous report found inclusion bodies only within the pneumocytes. Unfortunately, samples from this group of animals were not available to pursue adenovirus classification. Without confirmatory testing, viral etiology cannot be excluded, which remains a key limitation of our study. The pathogenic role of *M. pogonae* cannot be eluded until an experimental study fulfilling Koch’s postulate or a clinical report confirmed with *M. pogonae* as a sole pathogenic agent is available. One of the animals had marked intestinal parasitism (oxyurids and *Isospora* spp.) as a coinfection that is speculated to be the cause of the colitis/cloacitis found histologically.

This is the second report of *M. pogonae* in bearded dragons and represents a disease outbreak in a larger number of animals presenting with differences between host signalment from those in the first report [18]. All the animals in our research colony were juveniles in comparison to the adult bearded dragon reported by Crossland et al. [18]. In our study, animals died between 5 and 8 months old. The difference in age may imply that juveniles are susceptible to succumbing to disease from *M. pogonae* infection; however, more research on pathogenicity is required.

The source of *M. pogonae* is currently unknown. Animals had been shipped one week prior to the first death and underwent acclimatization to a new environmental and husbandry setting. The breeding animals from which our colony originated had been kept under seemingly appropriate husbandry conditions. However, the animals arrived with a wide weight range (4.1–28.2 g). For a 4-to-5-month-old bearded dragon, the weight of was considered low. The conditions of these juveniles suggest past husbandry issues and failure to thrive. We can only speculate that *M. pogonae* has been dormant and that stress from shipment and re-acclimatization to new conditions may have contributed to the expression of clinical signs. In our research setting, there was no basking area provided, as this would have elevated the overall temperatures in the enclosures. Even though an appropriate temperature gradient was provided, the lack of a basking area may have compromised thermoregulation, digestion, immune function, and metabolic activity. Coinfections with parasites were present in two of the animals. However, it is interesting to note that the single animal with inclusions was culture-negative for *M. pogonae* in lung tissue, but *M. pogonae* was recovered from the lung swab. Crossland et al. [18] stated that the role of *M. pogonae* in the disease process of the animal was unknown but speculated that the coinfection of both pathogens (*M. pogonae* and helodermatid adenovirus 2) may have exacerbated the disease, causing the animal to decline.

Most pathogenic mycoplasmal species are associated with chronic and clinically silent disease, resulting in high morbidity but low mortality exacerbated by stress; subclinical infections and intermittent shedding contribute to persistence in affected populations [32]. In chelonians, natural *M. agassizii* infections [33,34] and experimental infections to fulfill Koch’s postulates confirmed that *M. agassizii* causes URTD in both desert [4] and gopher [5] tortoises. A small-scale experimental infection confirmed that *M. testudineum* infection resulted in URTD but with less severe lesions than *M. agassizii* [35]. In crocodilian hosts, more severe clinical disease was found in *M. alligatoris* [36,37] and *M. crocodylii* [38,39] infections. Other mycoplasma species isolated from reptilian hosts appear to have limited to no pathogenic potential [40,41]. While it is a possibility that central bearded dragons harbor *M. pogonae* as a part of their normal microbiome, the detection of *M. pogonae* from the lower respiratory tract of animals with pneumonia during the mortality events strongly suggests that *M. pogonae* is indeed pathogenic. Further studies on *M. pogonae* are needed to establish a body of knowledge on this pathogen and its clinical implications.

Mycoplasmas rely on their host for nutrient scavenging, as they have lost many metabolic capabilities through evolutionary genome reduction [32]. As a result, growth media for axenic cultivation generally are extremely rich and complex. Because the cell wall has also been lost, most media contain a serum source to provide cholesterol as well as yeast extract. Additionally, fresh samples enhance the chance of isolation. If this is not possible, then freezing tissues and swabs in antibiotic-free transport solution containing growth medium or a protein source to stabilize the membrane is preferred, but some loss of variability can occur. To enhance viability, it is important that swabs are placed in a cryoprotective medium and not shipped dry. Initial isolation attempts for new species often require initial growth on multiple media, different serum sources, and different incubation temperatures. In cases where the 16S rRNA sequence is available from molecular testing, the closest related known species often can inform the best culture conditions to use for isolation attempts. To date, SP4 broth and agar has proven to be a reliable growth medium for mycoplasmas from reptilian hosts. A major consideration for *Mycoplasma* spp. is the incubation temperature, length of incubation, and colony size. *M. agassizii*, *M. alligatoris*, *M. insons*, and *M. testudineum* are temperature-restricted and grow at 30 °C. In contrast, *M. crocodyli*, *M. iguanae*, and *M. testudinis* can grow at higher temperatures. There is also variation in the time needed to see growth, ranging from 1 to 4 days (*M. alligatoris*, *M. crocodyli*, and *M. pogonae*) to see a color change in broth and colonies on agar being from 3 to 6 weeks (*M. agassizii* and *M. testudineum*), with colonies visible only with 40× magnification. While the difficulty, time needed, and experience needed to grow *Mycoplasma* spp. in axenic cultures of mycoplasmas is challenging, having pure cultures available for the scientific community as well as for whole-genome sequence analyses will be critical for unraveling the pathogenic potential of these microbes.

There has been an increasing recognition that WGS can be used for species determination for prokaryotes [28,30]. The use of WGS for the speciation of Phytoplasmas [29] also supports the use of WGS in defining a new species. *M. pogonae* meets the established molecular criteria [28,30] for a new species based on the 16S rRNA sequence as well as a whole-genome comparison at both the ANI and dDDH levels.

To date, there is no prevalent study of *M. pogonae*. The original detection of *M. pogonae* was through 16S rRNA sequencing of a captive adult bearded dragon with pneumonia in the USA. However, there are two *M. pogonae* 16S rRNA sequences reported from the nasal cavity and fistula of two bearded dragons in Europe; no information is provided as to the clinical condition of these animals. Our study is the first report of culture isolation of *M. pogonae* from the lower respiratory tracts of multiple juvenile bearded dragons during a mortality event. We also developed a *M. pogonae* species-specific PCR assay based on the *uvrA* gene.

## 5. Conclusions

Our results implicate *M. pogonae* infection as the cause of sudden mortality in a colony of captive central bearded dragons in the USA. The present study provides clinical and pathological characteristics of *M. pogonae* infection, which will aid in making a diagnosis of non-specific and upper respiratory-related clinical signs found in central bearded dragons. Furthermore, based on the findings of the study, a strong causal relationship was presumed between *M. pogonae* infection and the observed clinical signs/pathology, suggesting that *M. pogonae* is pathogenic and can act as a primary infectious agent. Finally, we used whole-genome sequencing to confirm that *M. pogonae* is a new mycoplasma species.

## Figures and Tables

**Table 1:** Clinical signs, culture isolation of *M. pogonae*, and pathology of the six mortality events *.

Animal ID	Date of Death	Sample Type	Culture	Tissue PCR	Sex	Weight	Clinical Signs	Macroscopic and Microscopic Findings
Case 1	8 August 2023	FFPE Scrolls	N/A	POS	Female	7.6 g	Lethargy, blepharospasm OU, inappetence	Emaciation, marked histiocytic pneumonia
Case 2 (L2313072 Type Strain for Genome)	19 August 2023	Lung Tissue	POS	POS	Male	8.4 g	Lethargy, weight loss, inappetence	Emaciation, lymphoplasmacytic and histiocytic pneumonia with marked pulmonary edema, mild conjunctivitis, nasopharyngitis, dacryocystitis, and tracheitis
Case 3	7 September 2023	Lung Tissue	POS	POS	Female	22 g	Lethargy, decreased appetite, cervical extension posture and sporadic open mouth breathing for 3 days	Histiocytic and granulocytic pneumonia, ulcerative colitis/cloacitis. Large numbers of *Isospora* sp. and oxyurid ova seen in colon
Case 4	15 September 2023	Trachea	POS	POS	Female	3.63 g	Lethargy, blepharospasm OU, inappetence, dehydration	Emaciation, mild celomic effusion, rhinitis, stomatitis, pharyngitis. Lymphohistiocytic inflammation of the liver, lung, upper respiratory tract, and oral cavity with numerous large intranuclear inclusion bodies within hepatocytes, pneumocytes, and colonic epithelial cells.
Case 5	12 October 2023	Lung Tissue Lung Swab	NEG POS	ND	Male	3.5 g	Lethargy, inappetence, stunted growth, dehydration >10%	Emaciation, necrotizing enterocolitis, hepatocellular necrosis, and harderian gland dacryoadenitis. Large intranuclear inclusion bodies in enterocytes, hepatocytes, and Harderian gland epithelial cells
Case 6	14 November 2023	Lung Tissue Lung Swab	POS POS	ND	Male	23.65 g	Lethargy, inappetence, weight loss, dehydration 5%	Emaciation, marked to severe heterophilic pneumonia, numerous intestinal parasitism suspected as *Balantidium* sp., and increased granulocyte production in bone marrow

* FFPE = formalin-fixed paraffin embedded; OU = *Oculus Uterque* (both eyes); POS = positive, NEG = negative; N/A = not applicable; ND = not done.

**Table 2:** Pairwise digital DNA/DNA hybridization (dDDH) values between *M. pogonae* and selected type strain genomes using the Type (Strain) Genome Server: https://tygs.dsmz.de.

*M. pogonae* L2313072 with Type Strains of	dDDH (d0, in %)	C.I. (d0, in %)	dDDH (d4, in %)	C.I. (d4, in %)	dDDH (d6, in %)	C.I. (d6, in %)	G+C Content Difference (in %)
*M. phocimorsus* M5725T	12.7	[10.0–15.9]	27.1	[24.7–29.6]	13.1	[10.7–15.8]	1.83
*M. miroungirhinis* ES2806-NAS	12.7	[10.0–16.0]	25.7	[23.4–28.2]	13.1	[10.8–15.9]	2.94
*M. ovipneumoniae* ATCC 29419	12.6	[9.9–15.9]	25.4	[23.0–27.8]	13	[10.7–15.8]	2.22
*M. agassizii* ATCC 700616	12.6	[9.9–15.9]	25.1	[22.8–27.6]	13	[10.7–15.8]	1.45
*M. alligatoris* ATCC 700619	12.7	[10.0–15.9]	24.9	[22.6–27.4]	13.1	[10.7–15.8]	0.32
*M. zalophi* CSL 4296	12.7	[10.1–16.0]	24	[21.7–26.5]	13.1	[10.8–15.9]	1.75
*M. bovis* PG45	12.7	[10.0–15.9]	24	[21.7–26.5]	13.1	[10.7–15.8]	2.31
*M. testudineum* ATCC 700618	12.7	[10.0–16.0]	23.2	[20.9–25.7]	13.1	[10.8–15.8]	0.55
*M. neophronis* DSM 24097	12.7	[10.0–15.9]	22.3	[20.0–24.7]	13.1	[10.7–15.8]	3
*M. neurolyticum* NCTC 10166	12.8	[10.1–16.1]	21.2	[19.0–23.6]	13.2	[10.9–16.0]	3.87
*M. canadense* 275c	12.7	[10.0–16.0]	21.1	[18.8–23.5]	13.1	[10.8–15.9]	2.67
*M. collis* ATCC 35278	12.8	[10.1–16.1]	20.9	[18.7–23.4]	13.2	[10.8–15.9]	4.59
*M. iguanae* 2327	13.4	[10.7–16.7]	20.9	[18.7–23.3]	13.7	[11.3–16.5]	0.06
*M. moatsii* ATCC 27625	12.8	[10.1–16.1]	20.8	[18.6–23.2]	13.2	[10.8–15.9]	0.55
*M. crocodyli* MP145	12.7	[10.1–16.0]	20.6	[18.4–23.0]	13.1	[10.8–15.9]	0.05
*M. procyoni* LR5794	13	[10.3–16.3]	20.4	[18.2–22.8]	13.4	[11.0–16.1]	2.02
*M. molare* ATCC 27746	13.1	[10.4–16.4]	20.1	[17.9–22.5]	13.5	[11.1–16.3]	2.13
*M. anserisalpingitidis* ATCC BAA-2147T	12.8	[10.1–16.0]	19.9	[17.7–22.3]	13.1	[10.8–15.9]	0.38
*M. hyorhinis* ATCC 17981	12.7	[10.1–16.0]	19.5	[17.3–21.9]	13.1	[10.8–15.9]	1.15
*M. lagogenitalium* 12MS	13.4	[10.6–16.7]	18.1	[15.9–20.4]	13.7	[11.3–16.5]	2.16
*M. testudinis* ATCC 43263	12.6	[9.9–15.9]	16.4	[14.3–18.7]	13	[10.7–15.7]	4.54

The dDDH values are provided along with their confidence intervals (CIs) for the three different GBDP formulas. The proposed and generally accepted species boundary for digital DNA/DNA hybridization (dDDH) values are 70%. Websites for the programs used in these analyses are https://www.ezbiocloud.net/tools/orthoaniu and https://tygs.dsmz.de.

## Data Availability

The original contributions presented in this study are included in the article. Further inquiries can be directed to the corresponding author.
